# Evaluation of partial body weight for predicting body weight and average daily gain in growing beef cattle

**DOI:** 10.1093/tas/txab126

**Published:** 2021-07-22

**Authors:** Michael D MacNeil, Donagh P Berry, Sam A Clark, John J Crowley, Michiel M Scholtz

**Affiliations:** 1 Delta G, 145 Ice Cave Rd., Miles City, MT 59301, USA; 2 Department of Animal, Wildlife and Grassland Sciences, University of the Free State, Bloemfontein 9300, South Africa; 3 Agricultural Research Council, Animal Production, Irene 0062, South Africa; 4 Teagasc, Animal & Grassland Research and Innovation Centre, Moorepark, Fermoy, Co. Cork, Ireland; 5 School of Environmental and Rural Science, University of New England, Armidale, New South Wales 2351, Australia; 6 AbacusBio Ltd., 442 Moray Place, Dunedin 9016, New Zealand; 7 Department of Agriculture, Food and Nutritional Science, University of Alberta, Edmonton, AB, Canada

**Keywords:** live weight, performance test, prediction, real-time data capture, test length

## Abstract

Information on body weight and average daily gain (ADG) of growing animals is key not only to monitoring performance, but also for use in genetic evaluations in the pursuit of achieving sustainable genetic gain. Accurate calculation of ADG, however, requires serial measures of body weight over at least 70 days. This can be resource intensive and thus alternative approaches to predicting individual animal ADG warrant investigation. One such approach is the use of continuously collected individual animal partial body weights. The objective of the present study was to determine the utility of partial body weights in predicting both body weight and ADG; a secondary objective was to deduce the appropriate length of test to determine ADG from partial body weight records. The dataset used consisted of partial body weights, predicted body weights and recorded body weights recorded for 8,972 growing cattle from a range of different breed types in 35 contemporary groups. The relationships among partial body weight, predicted body weight and recorded body weight at the beginning and end of the performance test were determined and calculated ADG per animal from each body weight measure were also compared. On average, partial body weight explained 90.7 ± 2.0% of the variation in recorded body weight at the beginning of the postweaning gain test and 87.9 ± 2.9% of the variation in recorded body weight at its end. The GrowSafe proprietary algorithm to predict body weight from the partial body weight strengthened these coefficients of determination to 95.1 ± 0.9% and 94.9 ± 0.8%, respectively. The ADG calculated from the partial body weight or from the predicted body weight were very strongly correlated (*r* = 0.95); correlations between these ADG values with those calculated from the recorded body weights were weaker at 0.81 and 0.78, respectively. For some applications, ADG may be measured with sufficient accuracy with a test period of 50 days using partial body weights. The intended inference space is to individual trials which have been represented in this study by contemporary groups of growing cattle from different genotypes.

## INTRODUCTION

Average daily gain (ADG) is an important component of production efficiency for growing beef cattle (Nielsen et al., 2013). The mathematical relationships among traits contributing to efficiency imply that for a pair of calves with the same initial body weight, the one with a faster ADG will reach a target market weight with fewer days on feed and thus could be more efficient due to the allocation of less feed to maintenance. Archer et al. (1997) and Marzocchi et al. (2020) found that a period of 70 days was needed to provide adequate precision in measuring the efficiency of growing calves. The primary reason for this length of time was to obtain a sufficiently precise measurement of ADG rather than feed intake. In contrast, Archer and Bergh (2000) indicated that a period of 42–56 days was adequate to measure ADG in growing calves. Kearney et al. (2004) also suggested that use of automated liveweight measurement would allow the duration of tests to be decreased to 56 days without reducing the precision of estimates of liveweight change. However, Culbertson et al. (2015) previously documented how a 56-day test period in growing beef cattle underestimates ADG. Therefore, the Beef Improvement Federation (2021) has adopted a 70-day test period as its de facto standard for measuring ADG in growing cattle.

Researchers have conjoined a weighing scale with a watering device to facilitate the recording body weight in real time (Currie et al., 1989). However, this apparatus did not achieve widespread use until GrowSafe developed a system to capture partial body weight in real time as a means of increasing the frequency of observations that can be used to predict body weight or “in-pen body weight” and ADG (Benfield et al., 2017). Multiple companies currently market systems that passively capture of body weight or partial body weight (Wang et al., 2021). Because bi-weekly weighing of animals on a 70-day test at approximately equally spaced intervals over the test period has been recommended to increase the accuracy of the ADG measurement (Crews and Carstens, 2012), more regular recording of body weight may also improve the accuracy of calculated ADG. In addition, processing animals through a conventional chute and scale system imposes some degree of stress (Cooke, 2014; Haskell et al., 2014; Lees et al., 2020) and is labor intensive. This stress and the associated labor costs (after safety concerns) can be averted if body weight can be measured without human intervention.

The objectives of the present study were to determine the utility of partial body weights in predicting both body weight and ADG as well as determine the appropriate length of test for predicting ADG from daily measures of partial body weight. The intended inference is to individual trials which have been represented in this study by contemporary groups of growing cattle.

## MATERIALS AND METHODS

### Data

Data for this study were provided by Vytelle Inc., the manufacturer of the GrowSafe equipment used to record partial body weights of growing beef animals. Vytelle captures these data in real time from equipment that has been installed at commercial and academic facilities internationally. Because the data arose from the database of an industry service provider, there was no prior evaluation of animal care and use for this particular study by an institutional animal care and use committee.

**Figure 1.**
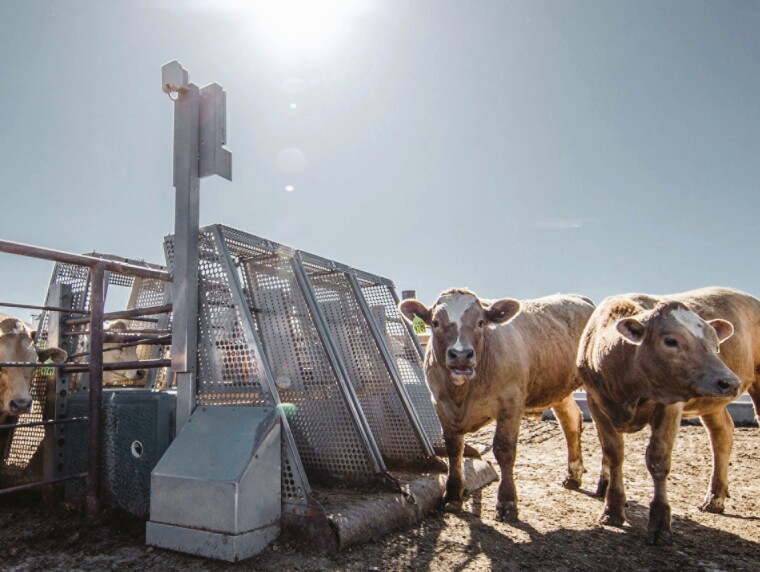
Operational system for the capture of partial body weight data from growing beef animals.

Benfield et al. (2017) described the technology used for the data collection. Briefly, a platform scale was positioned such that an individual animal must place its front feet on the scale in order to drink from a water trough (Figure 1). Multiple weighing units may be positioned adjacent to a single trough to allow more than one animal to drink at a time. The data used in the present study originated from 35 contemporary groups of beef animals from different genotypes on postweaning gain tests in various locations. The number of animals per contemporary group ranged from 4 to 123 and the duration of the test period was from 63 to 175 days. Bulls and heifers represented in the data were *Bos indicus* (Brahman), *Bos taurus africans* (Afrikaner and Nguni), *Bos Taurus* (Angus, Akaushi, Charolais, Hereford, Holstein, Red Angus, Salers, Simmental, and South Devon), various crosses with Angus, Afrikaner, Bonsmara, Nguni and Simmental, and Bonsmara and Simbrah composite animals. The data were collected between the years 2016 and 2020, inclusive.

The full data set consisted of daily partial body weight records from the GrowSafe Beef system records on 8,972 animals along with body weights recorded at the beginning and end of the test period as well as sporadically in between. The partial body weight used in this study was a summarization of the observations captured in real time for an animal on a given day, with only minimal editing to remove values that were obviously erroneous. From the partial body weight, a predicted body weight was generated using a proprietary algorithm. The recorded body weight was a measurement of body weight that was recorded a minimum of four times during each test by weighing each animal using a standard weighing scale.

### Analysis

The first analysis assessed the relationships between both the GrowSafe partial body weight and predicted body weight with the recorded body weight at the beginning of the test period (i.e., −2 <day<4). There were a few contemporary groups with no recorded body weights on or about day 0; these groups were not used in this assessment. The PROC MIXED procedure (SAS Institute Inc., Cary North Carolina, USA) was used to estimate parameters for the model:


$$\begin{array}{l} y_{ij}=g_{i}+b_{.}^{\prime }w_{ij}+b_{i}w_{ij}+e_{ij} \end{array}$$

with the equivalent model


$$\begin{array}{l} y_{ij}=g_{i}+b_{i}w_{ij}+e_{ij} \end{array}$$

used to estimate the contemporary group-specific regression equations. In the above equations $y_{ij}=$ the recorded body weight that was recorded for the *j*th animal at the beginning of the postweaning gain test; $g_{i}=$ the *i*th contemporary group in which the *j*th animal was fed; $w_{ij}=$ the partial body weight or predicted body weight of the *j*th animal; $b_{.}^{\prime }=$ the pooled regression coefficient of recorded body weight on either partial body weight or predicted body weight; $b_{i}=$ the partial regression coefficient of recorded body weight on either partial body weight or predicted body weight for the *i*th contemporary group (to be estimated); and $e_{ij}=$ the residual deviation of the recorded body weight from its predicted value for the *j*th animal in the *i*th contemporary group. Subsequent to the analysis of variance, estimates of the correlations among partial body weight, predicted body weight and recorded body weight were calculated. The regression coefficient of recorded body weight on predicted body weight was tested against the *a priori* expected value of 1.0. The difference between recorded body weight and the predicted body weight was tested against the *a priori* expected value of 0.0. Similar analyses were conducted for the three weight traits recorded at the end of each test.

Next, three measures of ADG for each animal were calculated by linear regression based on observations of partial body weight, predicted body weight and recorded body weight. The three measures of ADG were then subjected to an analysis of variance using PROC MIXED (SAS Institute Inc., Cary North Carolina, USA) with the following model:


$$\begin{array}{l} y_{ijk}=g{}_{i}+m_{j}+gm_{ij}+e_{ijk} \end{array}$$

wherein, $y_{ijk}=$ a measure of ADG for the *k*th animal; $g_{i}=$ the effect of the *i*th contemporary group (*i* = 1 to 35); $m_{j}$ = the effect of the *j*th measure of ADG (*j* = 1 to 3); $gm_{ij}$ = the effect of the interaction between the i^th^ contemporary group and *j*th measurement; and $e_{ijk}$ = the residual deviation of the ADG for the *k*th animal in the *i*th contemporary group calculated from the *j*th set of measurements from its predicted value. Within each contemporary group, the ADG calculated based on predicted body weight was contrasted with the value of ADG calculated from the recorded body weights. The correlations among the three measures of ADG were estimated within each contemporary group.

The test data from each contemporary group was truncated at 70 days on test as this reflects the standard length of test for measuring ADG with bi-weekly recorded body weights, against which evaluations of shorter duration were compared (Wang et al., 2006). The data for partial body weight were evaluated using a similar approach with and without imposing the criterion that the linear regression of weight on time (day) for each animal had an *R*^2^ ≥ 0.9 (J. Basarab, University of Alberta, personal communication, 18 March 2021). This requirement was established for body weights recorded at 14-day intervals to ensure linear growth during the test period without hindrance from morbidity or nutritional restrictions (Basarab et al., 2003). Imposing the requirement that the regression of weight on age for each animal had an *R*^2^ ≥ 0.9 over a test period of 61 to 70 days resulted in the loss of 38.4% of the partial body weight data. For individual contemporary groups the loss ranged from 0% to 98%. It could be that the random day-to-day fluctuations in partial body weight compromise the use of an *R*^2^ value as an editing criterion.

A grid search was initiated to estimate the appropriate length of test to determine ADG from daily partial body weight records. The starting point for the search was to split the data set at 35 days on test. The ADG calculated for the full test period was the dependent variable in a regression model that considered ADG calculated from the two parts of the test (i.e., the first 35 days and the latter 35 days) as independent variables. This regression analysis was done using sequential of Type 1 sums of squares such that the partial sum of squares for variation in full test period ADG explained by the ADG observed in the first period of the test was not adjusted for the ADG observed in the second period of the test. However, the partial sum of squares for variation in the full test period ADG explained by the ADG observed in the latter period of the test was adjusted for the ADG observed in the first period of the test. Subsequent to this analysis, the data for each animal was split at 50 days on test and the analysis was conducted again.

## RESULTS AND DISCUSSION

### Utility of Partial Body Weights

In analyses of variance for the assessment of partial body weight and predicted body weight as predictors of recorded body weight at the beginning of the test period, the interaction of the contemporary group with the regression effects were highly significant (*P* < 0.01). Partial body weight explained 73.4–99.5% of the variation in recorded body weight, while predicted body weight explained 86.5–99.6 % of the variation in recorded body weight (Table 1). Thus, it can be concluded that for greatest accuracy in the prediction of recorded body weight at the initiation of a postweaning gain test, the calibrations of the relationship between partial body weight and predicted body weight with recorded body weight should be specific to the contemporary group. In addition, relative to the regression of recorded body weight on partial body weight, the prediction of recorded body weight from predicted body weight increased the coefficient of determination from 0.91 to 0.95 and reduced the average standard error of the estimate from 0.19 to 0.07. For eight of the contemporary groups, the intercept of the regression equation for recorded body weight on partial body weight was positive (*P* < 0.05). Thus, for these contemporary groups using a multiplicative adjustment to predict recorded body weight (i.e., predicted body weight) from partial body weight would result in an under- and over- estimate of the recorded body weight for animals with above or below average partial body weight, respectively. There were no negative estimates of this intercept that approached significance (*P* > 0.10).

**Table 1.** Estimates per contemporary group (CG where the number of records is given by N) from the regression of recorded body weight on initial partial body weight and on predicted body weight, estimates of the correlations of partial and predicted body weight with the recorded body weight, and the mean difference between the recorded body weight and predicted body weight at the initiation of the test period

CG	N	Partial body weight		Predicted body weight			Difference (kg)
		*b* ± SE	*r*_1_	b ± SE	*b* = 1.0	*r*_2_	
1	32	1.738 ± 0.100	0.977	0.995 ± 0.0376	ns	0.989	−3.91 ± 2.01^†^
2	34	1.827 ± 0.129	0.983	1.099 ± 0.0510	^†^	0.992	4.78 ± 1.95^*^
3	43	1.497 ± 0.090	0.950	0.879 ± 0.0291	^**^	0.955	−9.91 ± 1.48^**^
5	20	1.833 ± 0.243	0.975	1.075 ± 0.0937	ns	0.978	−3.22 ± 2.55
6	20	1.737 ± 0.374	0.923	0.933 ± 0.1272	ns	0.956	−1.54 ± 2.55
7	26	1.773 ± 0.233	0.957	1.032 ± 0.0882	ns	0.974	−4.05 ± 2.23^†^
9	31	1.864 ± 0.188	0.985	1.067 ± 0.0708	ns	0.989	−2.94 ± 2.04
10	15	1.732 ± 0.178	0.992	0.995 ± 0.0677	ns	0.993	−1.44 ± 2.94
11	9	1.669 ± 0.334	0.946	1.008 ± 0.1205	ns	0.960	−0.39 ± 3.79
12	11	1.747 ± 0.182	0.995	1.089 ± 0.0748	ns	0.996	−1.98 ± 3.43
13	11	1.798 ± 0.319	0.971	1.106 ± 0.1279	ns	0.985	−7.68 ± 3.43^*^
14	4	1.404 ± 1.162	0.985	0.897 ± 0.4856	ns	0.996	3.72 ± 5.69
15	25	1.645 ± 0.216	0.976	1.000 ± 0.0861	ns	0.983	3.01 ± 2.28
16	5	1.669 ± 0.373	0.987	1.005 ± 0.1489	ns	0.986	−3.83 ± 5.09
17	23	1.621 ± 0.146	0.988	1.015 ± 0.0606	ns	0.987	0.73 ± 2.37
18	33	1.517 ± 0.147	0.954	1.045 ± 0.0759	ns	0.985	0.03 ± 2.01
19	97	1.617 ± 0.074	0.953	0.992 ± 0.0291	ns	0.983	16.27 ± 1.16^**^
20	56	1.720 ± 0.110	0.976	1.048 ±0.0442	ns	0.977	19.26 ± 1.52^**^
23	33	1.820 ± 0.086	0.985	1.008 ± 0.0314	ns	0.993	1.46 ± 1.40
24	70	1.517 ± 0.052	0.936	0.905 ± 0.0198	^**^	0.977	29.61 ± 0.96^**^
25	114	0.849 ± 0.037	0.734	0.843 ± 0.0196	^**^	0.921	13.53 ± 0.76^**^
26	44	1.425 ± 0.063	0.909	0.959 ± 0.0262	ns	0.971	13.42 ± 1.21^**^
27	47	1.612 ± 0.061	0.856	0.919 ± 0.0228	^**^	0.865	3.09 ± 1.17^**^
28	91	0.946 ± 0.037	0.771	0.899 ± 0.0188	^**^	0.961	10.20 ± 0.85^**^
29	102	1.735 ± 0.110	0.972	1.016 ± 0.0419	ns	0.986	−3.39 ± 1.13^**^
30	57	1.693 ± 0.068	0.993	0.989 ± 0.0261	ns	0.992	11.55 ± 1.51^**^
31	123	1.754 ± 0.071	0.975	0.960 ± 0.0255	ns	0.980	4.09 ± 1.03^**^
32	26	1.587 ± 0.132	0.978	0.997 ± 0.0543	ns	0.990	10.30 ± 2.23^**^
33	58	1.817 ± 0.169	0.980	1.022 ± 0.0629	ns	0.977	−2.78 ± 1.49^†^

ns *P* ≥ 0.10, ^†^*P* < 0.10, ^*^P < 0.05, ^**^P < 0.01 testing the null hypothesis that the regression of predicted body weight on recorded body weight = 1.0, and for testing the null hypothesis that the difference between recorded body weight and predicted body weight = 0.0.

The pooled regression coefficient of recorded body weight on partial body weight was 1.82 ± 0.15 across all contemporary groups studied (Table 1). In contrast, Benfield et al. (2017) indicated that body weight in cattle may be estimated as partial body weight multiplied by a constant of 1.677 across sexes and breed types. The 95% confidence interval for the Benfield et al (2017) estimate was approximately 1.3–2.1. All of the estimates obtained for the regression of recorded body weight on initial partial body weight in the present study fell within that range and the pooled regression coefficient estimated here is also not significantly different from that proposed by Benfield et al. (2017). For seven of the 29 contemporary groups in the present study, the partial body weight explained less than 90% of the variation in recorded body weight at the initiation of the test period. These coefficients of determination may be interpreted as indicating anomalous relationships between the recorded body weight and the partial body weight. The remarkably unusual regression coefficients relating partial body weight and recorded body weight that were obtained for contemporary groups 25 and 28 were due to a few erroneous observations of partial body weight in each of these two contemporary groups indicating a clear need for careful data management.

Also shown in Table 1 are estimates from the regression analyses of recorded body weight on predicted body weight as well as the mean difference between the recorded body weight and predicted body weight at the initiation of the test period. The pooled regression of predicted body weight on recorded body weight was 1.02 ± 0.05. For five of the contemporary groups (i.e., numbers 3, 24, 25, 27, 28), the regression of predicted body weight on the recorded body weight differed (*P* < 0.01) from 1.0 which is its *a priori* expected value. Compared to the prediction of recorded body weight from partial body weight, the prediction of recorded body weight from the predicted body weight generated by the GrowSafe proprietary algorithms explained, on average, 4.4% more of the variation in recorded body weight. This outcome was anticipated because the prediction algorithm would presumably reduce the random variability in body weight compared to that which was present in the partial body weights. Positive (negative) values for the difference between the recorded body weight and predicted body weight indicate the recorded body weight was, on average, greater (less) than the predicted body weight. These disparities between the predicted body weight and recorded body weight differed from zero for approximately half the contemporary groups. It should be recognized that this mean difference in body weight per contemporary group does not necessarily reflect bias because both harbor systematic error in their respective recording. Such error could include human error, scale calibration, or indeed the effects of gut fill.

In the analyses of variance for the assessment of partial body weight or predicted body weight as predictors of recorded body weight at the end of the test period, the interaction of contemporary group effects with the regression coefficients were also significant (*P* < 0.01). Thus, as for the initial weight, it can be concluded that for greatest accuracy in the prediction of recorded body weight at the end of a postweaning gain test, the calibration depicting the relationship between partial body weight and predicted body weight with recorded body weight should be estimated per contemporary group. Because the regression coefficients for recorded body weight on predicted body weight at the beginning and end of the test period are not perfectly correlated (*r* = 0.77) implies changes in the prediction model coefficients are needed as the test progresses.

Estimates of the regression coefficients of recorded body weight on partial body weight recorded at the end of the test period are summarized in Table 2. The pooled regression of partial body weight at the end of test on recorded body weight was 1.73 ± 0.09. While this value is again numerically greater than the constant multiplier suggested by Benfield et al (2017), it is not significantly different from that value. It is also not different from the regression of 1.82 estimated in the present study for the initiation of test. Relative to the regression of recorded body weight on partial body weight, the prediction of recorded body weight from predicted body weight increased the coefficient of determination from 0.85 to 0.95 and reduced the average standard error of the estimate from 0.20 to 0.06.

**Table 2.** Estimates per contemporary group (CG where the number of records is given by N) from the regression analyses of recorded body weight on initial partial body weight and predicted body weight, estimates of the correlations of partial and predicted body weight with the recorded body weight, and the mean difference between the recorded body weight and predicted body weight at the end of the test period

CG	N	Partial body weight		Predicted body weight			Difference (kg)
		*b* ± SE	*r*_1_	*b* ± SE	*b* = 1.0	*r*_2_	
1	32	1.702 ± 0.111	0.989	0.993 ± 0.041	ns	0.990	−5.16 ± 2.17^*^
2	34	1.895 ± 0.151	0.989	1.101 ± 0.055	^†^	0.992	3.97 ± 2.11^†^
3	43	1.527 ± 0.075	0.954	0.987 ± 0.021	ns	0.970	0.29 ± 1.87^ns^
4	54	1.588 ± 0.083	0.969	0.952 ± 0.031	ns	0.983	−3.50 ± 1.67^*^
5	22	1.838 ± 0.280	0.964	0.965 ± 0.049	ns	0.991	−5.80 ± 2.62^*^
6	20			0.775 ± 0.051	^**^	0.943	−0.45 ± 2.75^ns^
7	26	1.773 ± 0.265	0.957	1.032 ± 0.096	ns	0.974	−4.05 ± 2.41^†^
8	16	1.688 ± 0.265	0.988	1.046 ± 0.050	ns	0.990	3.85 ± 3.07^ns^
9	27	1.771 ± 0.358	0.957	1.078 ± 0.050	ns	0.962	−9.63 ± 2.37^**^
10	15	1.653 ± 0.147	0.991	1.006 ± 0.057	ns	0.988	0.29 ± 3.17^ns^
11	9	1.669 ± 0.380	0.946	1.008 ± 0.131	ns	0.960	−0.39 ± 4.10^ns^
12	11	1.747 ± 0.206	0.995	1.089 ± 0.081	ns	0.996	−1.98 ± 3.71^ns^
13	11	1.798 ± 0.362	0.971	1.106 ± 0.139	ns	0.985	−7.68 ± 3.71^*^
14	4	1.465 ± 0.925	0.930	0.815 ± 0.319	ns	0.946	6.64 ± 6.14^ns^
15	25	1.746 ± 0.128	0.994	1.056 ± 0.049	ns	0.992	8.17 ± 2.46^**^
16	5	1.545 ± 0.244	0.997	0.972 ± 0.097	ns	0.998	−2.11 ± 5.50^ns^
17	23	1.571 ± 0.126	0.981	0.971 ± 0.049	ns	0.985	3.01 ± 2.56^ns^
18	33	1.632 ± 0.128	0.977	1.005 ± 0.050	ns	0.983	4.37 ± 2.14^*^
19	98	0.792 ± 0.057	0.569	0.977 ± 0.025	ns	0.977	16.69 ± 1.24^**^
20	56	1.711 ± 0.114	0.978	1.034 ± 0.043	ns	0.982	17.93 ± 1.64^**^
23	23	1.324 ± 1.064	0.830	1.117 ± 0.044	^*^	0.983	0.325 ± 2.17^ns^
24	71	1.437 ± 0.088	0.960	0.907 ± 0.020	^**^	0.956	28.75 ± 1.47^**^
25	114	0.787 ± 0.057	0.712	0.843 ± 0.030	^**^	0.926	12.86 ± 1.15^**^
26	44	0.778 ± 0.074	0.690	0.966 ± 0.041	ns	0.972	12.85 ± 1.85^**^
27	47	1.647 ± 0.101	0.854	0.923 ± 0.035	^*^	0.867	3.04 ± 1.79^ns^
28	91	1.064 ± 0.063	0.806	0.885 ± 0.028	^**^	0.963	9.86 ± 1.29^**^
29	102	1.794 ± 0.091	0.983	1.025 ± 0.033	ns	0.982	3.20 ± 1.22^**^
30	57	1.681 ± 0.077	0.990	0.982 ± 0.028	ns	0.990	11.59 ± 1.63^**^
31	123	1.791 ± 0.063	0.984	1.000 ± 0.022	ns	0.987	4.60 ± 1.11^**^
32	26	1.533 ± 0.130	0.981	0.937 ± 0.050	ns	0.986	8.46 ± 2.41^**^
33	58	1.789 ± 0.172	0.976	1.041 ± 0.056	ns	0.981	−2.77 ± 1.61^†^
34	39	1.546 ± 0.066	0.986	0.914 ± 0.025	^**^	0.982	−4.65 ± 1.97^*^
35	31	1.726 ± 0.095	0.980	1.001 ± 0.034	ns	0.984	−2.07 ± 2.21^ns^

ns *P* ≥ 0.10, ^†^*P* < 0.10, ^*^P < 0.05, ^**^P < 0.01 testing the null hypothesis that the regression of predicted body weight on recorded body weight = 1.0, and for testing the null hypothesis that the difference between the recorded body weight and predicted body weight= 0.0.

The fact that separate scaling factors are required per contemporary group is in direct contrast to the conclusion of Benfield et al. (2017) who stated that body weight may be estimated as a constant multiple of partial body weight across sexes and breed types with adequate accuracy and precision. Use of a multiplicative constant to predict recorded body weight from a partial body weight assumes that the regression of body weight on partial body weight is linear and that the line it represents passes through the origin. The latter assumption is violated for contemporary groups 3 (*P* < 0.01), 19 (*P* < 0.01), 24 (*P* < 0.01), 25 (*P* < 0.01), 26 (*P* < 0.01), 28 (*P* < 0.01), 32 (*P* = 0.05), and 34 (*P* = 0.03). Thus, for these contemporary groups, the use of a constant multiplier weight would result in underestimation (overestimation) of recorded body weight when the y-axis intercept was positive (negative). Partial body weight is measured with the front feet on a scale and the hind feet on the ground. While this is not exactly comparable to weighing the front quarters of the animal, it should be noted that within- and across-breed variation exists in the relative weights of fore- and hind quarters of beef animals (Keane et al., 2011). This variation potentially contributes to the residual variance from the prediction of body weight from partial body weight.

It has long been recognized that daily variation in body weight of beef cattle exists (Lush et al., 1928). Hughes (1976) reported that content of the digestive tract is in constant flux and may weigh upwards of 25% of total body weight. Following the weighing of 1,018 growing cattle (mean age of 17 months) on 2 consecutive days, Kelly et al. (2019) reported a difference in body weight per animal between both days to be in the range of −18 to 22 kg with a standard deviation of 5.34 kg. The aforementioned differences were attributed primarily to variation in gut fill. Thus, the change in mass of the digesta can be one factor that results in short-term inaccuracy in the measurement of body weight which cannot be overcome by weighing the animals multiple times. Furthermore, the body weight of beef cattle changes with the time of day when they are weighed (Hughes, 1976; Heitschmidt, 1982) as well as the prevailing weather conditions at the time of weighing (Lush et al., 1928; Hughes, 1976). The degree to which these effects can be mitigated by the real-time regular capture of body weight is unknown. However, the complexity of the resulting data structure may suggest the opportunity to employ advanced analytical approaches like machine learning to predict the body weight of individual animals (Wang et al., 2021).

A potential application of the technology for passive collection of partial body weight is the capture of an age-constant measure of weight. For example, a measure of weight at 365-days could be recorded at exactly that age obviating the need for interpolation from weight records collected at flanking ages. Several phenotypes and estimated breeding values, such as residual feed intake (Koch et al., 1963) and residual intake and gain (Berry and Crowley, 2012), can also be calculated directly using the partial body weights. One complicating factor that needs to be considered in future research is how contemporary groups are defined for these age-specific measures. There may well be temporary environmental effects that differ between animals that are relatively close in age. In addition, the partial body weight or the ADG in partial body weight could be incorporated into multi-trait genetic evaluation directly as a correlated trait without the need to actually predict body weight from it. The best way to combine the different data sources into genetic evaluations is predicted on estimates of the genetic, residual and permanent environmental variance components for predicted and recorded body weights. A strong genetic correlation between the different measures is expected although a larger residual variance in predicted body weight may materialize. Therefore, an alternative strategy could be to combine the predicted and recorded body weights into a single trait weighting them differently based on the estimated variance components for each measure.

### Predicting ADG From Partial Body Weights

Summary statistics comparing the estimates of ADG from partial body weight or predicted body weight recorded daily throughout the test period versus ADG calculated from recorded body weight recorded periodically throughout the test period are in Table 3. As might be expected, the correlations of ADG estimated from partial body weight with ADG estimated from the predicted body weight (i.e., predicted from partial the body weight data) were generally very strong ($\bar{r}=\;0.96$) with only one contemporary group in which this correlation was < 0.8. The estimated correlations of recorded body weight with partial body weight and predicted body weight were weaker, averaging 0.81 ± 0.03 and 0.78 ± 0.04, respectively.

**Table 3.** Estimates per contemporary group (CG) of average daily gain calculated by linear regression of the measures of weight on test day along with the correlations among measures of ADG estimated from different sources of information, and a test of the mean difference between ADG estimated from recorded body weights and from predicted body weights

CG	Mean ADG from three measures of weight			Correlations among ADG values			Difference (kg)
	Predicted body weight (FW)	Partial weight (PW)	Recorded body weight (CW)	FW, PW	FW, CW	PW, CW	
1	1.705 ± 0.038	1.004 ± 0.038	1.283 ± 0.038	0.993	0.763	0.791	0.422 ± 0.054^**^
2	1.265 ± 0.037	0.733 ± 0.037	1.309 ± 0.037	0.993	0.866	0.869	−0.044 ± 0.052^ns^
3	1.665 ± 0.033	1.007 ± 0.033	1.778 ± 0.033	0.980	0.827	0.851	−0.113 ± 0.047^*^
4	1.914 ± 0.029	1.115 ± 0.029	1.743 ± 0.029	0.990	0.783	0.803	0.171 ± 0.042^**^
5	1.973 ± 0.046	1.154 ± 0.046	1.901 ± 0.046	0.996	0.951	0.949	0.072 ± 0.065^ns^
6	1.662 ± 0.048	0.960 ± 0.048	1.667 ± 0.048	0.993	0.857	0.869	−0.006 ± 0.068^ns^
7	1.613 ± 0.042	0.940 ± 0.042	1.582 ± 0.042	0.996	0.949	0.958	0.031 ± 0.060^ns^
8	1.608 ± 0.054	1.002 ± 0.054	1.656 ± 0.054	0.995	0.905	0.905	−0.048 ± 0.077^ns^
9	1.641 ± 0.039	0.946 ± 0.039	1.621 ± 0.039	0.998	0.962	0.958	0.020 ± 0.055^ns^
10	1.734 ± 0.056	1.039 ± 0.056	1.734 ± 0.056	0.990	0.933	0.895	−0.000 ± 0.079^ns^
11	1.702 ± 0.072	1.031 ± 0.072	1.782 ± 0.072	0.999	0.983	0.982	−0.080 ± 0.102^ns^
12	1.527 ± 0.065	0.930 ± 0.065	1.678 ± 0.065	0.997	0.969	0.975	−0.151 ± 0.092^ns^
13	1.421 ± 0.065	0.859 ± 0.065	1.474 ± 0.065	0.998	0.910	0.910	−0.053 ± 0.092^ns^
14	1.829 ± 0.108	1.109 ± 0.108	1.867 ± 0.108	0.974	0.880	0.940	−0.038 ± 0.153^ns^
15	1.767 ± 0.043	1.071 ± 0.043	1.830 ± 0.043	0.994	0.970	0.962	−0.063 ± 0.061^ns^
16	1.677 ± 0.097	1.011 ± 0.097	1.626 ± 0.097	0.999	0.988	0.986	0.051 ± 0.137^ns^
17	1.612 ± 0.045	0.978 ± 0.045	1.587 ± 0.045	0.999	0.957	0.951	0.025 ± 0.064^ns^
18	1.598 ± 0.038	0.972 ± 0.038	1.596 ± 0.038	0.999	0.963	0.966	0.002 ± 0.053^ns^
19	1.397 ± 0.022	0.771 ± 0.022	1.306 ± 0.022	0.939	0.787	0.822	0.091 ± 0.031^**^
20	1.476 ± 0.029	0.686 ± 0.029	1.429 ± 0.029	0.504	0.494	0.834	0.047 ± 0.041^ns^
21	1.533 ± 0.044	0.863 ± 0.044	1.248 ± 0.044	0.976	0.686	0.668	0.285 ± 0.062^**^
22	1.947 ± 0.032	1.098 ± 0.032	1.452 ± 0.032	0.940	0.480	0.457	0.495 ± 0.046^**^
23	1.068 ± 0.038	0.575 ± 0.038	0.854 ± 0.038	0.818	0.751	0.723	0.214 ± 0.053^**^
24	1.520 ± 0.026	0.819 ± 0.026	1.513 ± 0.026	0.836	0.107	0.268	0.007 ± 0.036^ns^
25	2.010 ± 0.020	1.182 ± 0.020	1.392 ± 0.020	0.921	0.371	0.473	0.618 ± 0.029^**^
26	1.987 ± 0.033	1.164 ± 0.033	1.394 ± 0.033	0.893	0.493	0.608	0.593 ± 0.046^**^
27	1.967 ± 0.032	1.083 ± 0.032	1.336 ± 0.032	0.905	0.463	0.644	0.631 ± 0.045^**^
28	1.981 ± 0.023	1.155 ± 0.023	1.384 ± 0.023	0.871	0.627	0.719	0.597 ± 0.032^**^
29	0.643 ± 0.021	0.372 ± 0.021	0.759 ± 0.021	0.998	0.932	0.930	−0.115 ± 0.030^**^
30	1.450 ± 0.029	0.861 ± 0.029	1.509 ± 0.029	0.977	0.832	0.843	−0.059 ± 0.041^ns^
31	1.635 ± 0.020	0.925 ± 0.020	1.505 ± 0.020	0.996	0.892	0.892	0.131 ± 0.028^**^
32	1.496 ± 0.042	0.852 ± 0.042	1.407 ± 0.042	0.994	0.923	0.911	0.089 ± 0.060^ns^
33	0.891 ± 0.028	0.515 ± 0.028	0.941 ± 0.028	0.999	0.811	0.814	−0.049 ± 0.040^ns^
34	1.094 ± 0.035	0.635 ± 0.035	1.012 ± 0.035	0.993	0.519	0.538	0.082 ± 0.049^†^
35	1.123 ± 0.039	0.654 ± 0.039	0.965 ± 0.039	0.994	0.738	0.744	0.158 ± 0.055^**^

ns *P* ≥ 0.10, ^†^*P* < 0.10, ^*^P < 0.05, ^**^P < 0.01 testing the null hypothesis that the difference = 0.0.

For 15 of the 35 contemporary groups, the mean ADG calculated from recorded body weights differed (*P* < 0.10) from the mean ADG calculated from predicted body weights (Table 3). The values derived from predicted body weight generally being greater than those from recorded body weight. These differences may, however, be due to imprecision in either method of weighing the animals.

### Length of Test When ADG is Predicted From Partial Body Weight

Many previous studies which attempted to determine the required minimum length of the test period (e.g., Wang et al., 2006; Culbertson et al., 2015; Ahlberg et al., 2018) for determining ADG made use of the part-whole correlation that has an asymptotic value of 1.0 as the length of the shortened test period approached the length of the full test period. This approach will invariably find that a shorter test period will explain nearly all of the variation in the full-length test. In the present study, a different approach based on model-order dependent regression, was used. In this approach, the early part of the test preceded the later part when specifying the independent variables in the regression model. Thus, the sum of squares due to regression for the first part of the test was not adjusted for the latter part of the test, but the sum of squares due to regression for the latter part of the test was adjusted for the portion of the test that preceded it. Thus, results for the early part of the test are exactly analogous to those obtained in the previous studies, while the results for the latter part of the test document the information that is lost when the test is reduced in length. Results for the regression analysis when the test period was split approximately in half (35 days on test) are presented in Table 4. Tests of greater than 70 days duration were truncated at 70 days.

**Table 4.** Results from the regression analyses per contemporary group (CG) relating average daily gain of partial body weight in two 35-day parts of the postweaning gain test to average daily gain during the full test period of approximately 70 days without prior editing to insure linearity of growth over the test period

CG	Length of test, *d*	Full test period		35-day test –w/o editing based on *R*^2^					
		*R*^2^, %	MS error, kg^2^	*b*_1_	*b*_2_	*R*^2^, %	partial *R*^2^, %	deviation (*d*)	Probability (*d* = 0)
1	63	88.8	6.326	1.24 ± 0.05	0.83 ± 0.04	53.4	51.9	−9.05 ± 1.32	<0.001
2	63	95.4	2.889	0.79 ± 0.03	0.62 ± 0.02	78.6	45.2	−1.69 ± 0.49	0.002
3	70	91.8	5.742	0.80 ± 0.05	1.06 ± 0.05	34.7	61.8	8.05 ± 1.40	<0.001
4	73	95.2	5.180	1.18 ± 0.03	0.93 ± 0.03	40.5	31.2	−1.19 ±0 .87	0.177
5	124	99.3	3.740	1.31 ± 0.03	1.14 ± 0.03	71.8	61.3	−1.47 ± 0.61	0.020
6	124	98.4	4.379	1.11 ± 0.03	0.96 ± 0.02	73.1	60.6	−3.05 ± 0.75	0.001
7	175	98.0	5.943	1.14 ± 0.04	0.99 ± 0.03	81.9	83.9	−2.76 ± 0.64	<0.001
8	76	88.4	7.479	0.96 ± 0.07	1.24 ± 0.08	36.0	77.0	0.87 ± 2.23	0.689
9	123	99.0	3.560	1.09±0.03	0.95 ± 0.02	91.1	63.1	−3.01 ± 0.48	<0.001
10	81	98.9	2.463	1.04 ± .03	1.01 ± 0.05	75.6	71.9	−0.21 ± 0.77	0.786
11	113	98.3	4.703	0.86 ± 0.05	1.10 ± 0.11	53.8	92.5	6.25 ± 1.77	0.008
12	113	97.4	5.313	0.76 ± 0.06	1.03 ± 0.08	65.1	82.0	5.45 ± 1.44	0.004
13	113	97.8	4.400	0.69 ± 0.05	0.91 ± 0.06	4.3	73.6	5.81 ± 2.08	0.019
14	99	99.3	3.023	1.08 ± 0.07	1.02 ± 0.07	59.2	96.1	2.15±2.28	0.415
15	99	99.1	3.249	0.95 ± 0.04	1.11 ± 0.04	81.7	77.6	5.33±0.91	<0.001
16	88	98.8	3.421	1.10 ± 0.09	0.94 ± 0.09	86.0	85.5	−1.61±2.03	0.472
17	88	98.7	3.322	1.07 ± 0.04	0.90 ± 0.03	90.5	69.1	−2.31±0.60	0.001
18	88	98.7	3.171	1.03 ± 0.02	0.94 ± 0.03	86.2	66.0	−1.53±0.49	0.004
19	64	70.7	10.806	0.81 ± 0.02	0.76 ± 0.02	57.2	40.0	−1.08±0.59	0.070
20	76	57.3	14.424	0.96 ± 0.03	0.71 ± 0.02	49.5	53.0	−4.22 ± 0.73	<0.001
21	74	80.8	7.529	0.51 ± 0.04	0.81 ± 0.04	21.4	58.1	13.16 ± 1.18	<0.001
22	74	87.9	7.514	1.29 ± 0.04	1.00 ± 0.03	11.5	47.9	−4.09 ± 1.31	0.003
23	71	81.2	5.561	0.72 ± 0.02	0.04 ± 0.02	80.1	33.4	−5.63 ± 0.48	<0.001
24	71	68.9	11.474	1.10 ± 0.02	0.86 ± 0.05	28.1	66.4	−11.21 ± 0.79	<0.001
25	61	87.8	7.546	1.35 ± 0.04	1.37 ± 0.03	63.4	5.3	−6.74 ± 0.82	<0.001
26	61	92.7	5.536	1.29 ± 0.05	1.20 ± 0.05	77.3	6.8	−4.47 ± 1.04	<0.001
27	61	92.7	5.478	1.13 ± 0.02	1.12 ± 0.04	42.4	46.4	−1.87 ± 0.67	<0.008
28	61	90.5	6.390	1.28 ± 0.04	1.26 ± 0.03	67.7	8.1	−4.91 ± 0.93	<0.001
29	72	85.0	2.635	0.30 ± 0.02	0.44 ± 0.01	63.5	68.0	2.58 ± 0.41	<0.001
30	73	88.5	6.220	0.89 ± 0.03	0.88 ± 0.03	38.1	58.8	−1.40 ± 0.90	0.121
31	88	94.9	5.234	0.89 ± 0.02	0.98 ± 0.02	31.9	71.6	1.57 ± 0.52	0.003
32	88	93.9	5.502	0.91 ± 0.03	0.73 ± 0.03	41.9	49.5	−2.01 ± 0.80	0.019
33	67	94.4	2.206	0.45 ± 0.02	0.67 ± 0.02	59.6	49.9	1.85 ± 0.43	<0.001
34	70	75.3	6.854	0.88 ± 0.06	0.66 ± 0.03	54.4	12.7	−9.58 ± 1.69	<0.001
35	70	79.8	6.117	0.83 ± 0.05	0.70 ± 0.04	34.0	33.5	−7.12 ± 1.78	0.001

The results in Table 4 pertaining to the full test period indicate the degree to which growth was linear over time. The regression coefficients *b*_1_ and *b*_2_ reflect the ADG from predicted body weight from day 1 to day 35 of the test and from day 36 to the end of the test, respectively. The *R*^2^ value is the coefficient of determination for the period from day 1 to day 35, and the partial *R*^2^ indicates the value of the information from the latter part of the test in determining ADG across the full test. The deviation, which has an expected value of 0.0, indicates the difference between the prediction of final partial body weight from its regression on days on test during the first period of the test and its recorded value at the end of the test period. Estimates of ADG from the initial period of the test explained more than 90% of the variation in ADG across the full test period for only two contemporary groups (i.e., groups nine and 17). Furthermore, in only three contemporary groups did the ADG recorded in the latter portion of the test contribute less than 10% to the measurement of full test ADG. Overall, the first and second half of the full test period explained a similar proportion of the variation in ADG across the full test period (*R*^2^ = 56.7 ± 3.8 vs. 56.0 ± 4.0, respectively). In an analysis that included all contemporary groups fitted as fixed effects, ADG in the first half of the test period explained 51.5% of the within contemporary group variance. The ADG from the latter half of the test period explained 33.1% of the within contemporary group variance after accounting for ADG during the first half of the test. For only six of the 35 contemporary groups, the prediction of final partial body weight based on the first part of the test not different (*P* > 0.10) from its observed value.

Imposing the requirement that the regression of partial body weight on age for each animal had to have an *R*^2^ ≥ 0.9 over a test period of 1–70 days to ensure the linearity of growth resulted in the loss of 38.4% of the partial body weight data (Table 5). Five contemporary groups were removed from the data set as a result of them containing fewer than four animals after this edit was imposed. It should be cautioned that this threshold was established based on weighing animals at two-week intervals throughout the test period (Crews and Carstens, 2012) rather than from daily weights. Again, after requiring each individual animal to exhibit approximately linear growth across the test period, the first and second half of the full test period explained, on average, a similar proportion of the variation in the ADG of partial body weight across the full test period (*R*^2^ = 60.0 ± 4.3 vs. 56.9 ± 4.4, respectively). In 20 of the 30 contemporary groups, the mean deviation of predicted partial body weight from observed mean body weight at the end of the test period was significant.

**Table 5.** Results from regression analysis per contemporary group (CG) relating average daily gain during the full test period of approximately 70 days to two 35-day periods of the postweaning gain test after editing the data to insure linearity of growth over the test period^1^

CG	Length of test, *d*	Full test period		35-day test –w/edit					
		*R*^2^, %	MS error (kg^2^)	*b*_1_	*b*_2_	*R*^2^, %	partial *R*^2^, %	deviation (*d*)	Probability (*d* = 0)
1	63	88.8	6.33	1.16 ± 0.06	0.87 ± 0.06	78.7	32.2	−4.00 ± 1.13	0.003
2	63	95.4	2.89	0.80 ± 0.02	0.62 ± 0.02	76.8	44.2	−1.91 ± 0.45	<0.001
3	70	91.8	5.74	0.88 ± 0.05	1.09 ± 0.05	37.4	66.0	6.54 ± 1.59	<0.001
4	73	95.2	5.18	1.18 ± 0.03	0.95 ± 0.03	43.9	28.5	−1.20 ± 0.90	0.189
5	124	99.3	3.74	1.31 ± 0.03	1.14 ± 0.03	71.8	61.3	−2.47 ± 0.62	0.001
6	124	98.4	4.38	1.11 ± 0.03	0.96 ± 0.02	73.1	60.6	−3.05 ± 0.75	0.001
7	175	98.0	5.94	1.14 ± 0.04	0.99 ± 0.03	81.9	83.9	−2.76 ± 0.64	<0.001
8	76	88.4	7.48	1.17 ± 0.08	1.14 ± 0.06	66.9	16.2	−4.38 ± 1.87	0.033
9	123	99.0	3.56	1.09 ± 0.03	0.95 ± 0.03	91.1	63.1	−3.01 ± 0.48	<0.001
10	81	98.9	2.46	1.04 ± 0.03	1.02 ± 0.04	75.6	71.9	−0.21 ± 0.77	0.789
11	113	98.3	4.70	0.86 ± 0.05	1.10 ± 0.11	53.8	92.5	6.25 ± 1.77	0.008
12	113	97.4	5.31	0.76 ± 0.06	1.03 ± 0.08	65.1	82.0	5.45 ± 1.44	0.004
13	113	97.8	4.40	0.69 ± 0.05	0.91 ± 0.06	4.3	73.6	5.82 ± 2.08	0.019
14	99	99.3	3.02	1.08 ± 0.07	1.02 ± 0.07	59.2	96.1	2.15 ± 2.28	0.415
15	99	99.1	3.25	0.95 ± 0.04	1.11 ± 0.04	81.7	77.6	5.32 ± 0.91	<0.001
16	88	98.8	3.42	1.10 ± 0.09	0.94 ± 0.09	86.0	85.5	−1.61 ± 2.03	0.472
17	88	98.7	3.32	1.07 ± 0.04	0.90 ± 0.03	90.5	69.1	−2.31 ± 0.60	0.001
18	88	98.7	3.17	1.03 ± 0.02	0.94 ± 0.03	86.2	66.0	−1.53 ± 0.49	0.004
19	64	70.7	10.81						
20	76	57.3	14.42	0.94 ± 0.03	0.76 ± 0.02	58.6	62.4	−3.08 ± 0.84	0.001
21	74	80.8	7.53						
22	74	87.9	7.51	1.26 ± 0.06	1.07 ± 0.04	4.7	42.1	−1.46 ± 2.09	0.487
23	71	81.2	5.56						
24	71	68.9	11.47						
25	61	87.8	7.55	1.30 ± 0.03	1.36 ± 0.04	66.7	11.7	−2.68 ± 0.63	<0.001
26	61	92.7	5.54	1.22 ± 0.03	1.13 ± 0.05	64.2	22.4	−2.80 ± 0.72	0.001
27	61	92.7	5.48	1.14 ± 0.03	1.12 ± 0.04	55.8	43.8	−1.93 ± 0.64	0.005
28	61	90.5	6.39	1.23 ± 0.03	1.21 ± 0.03	67.8	29.8	−2.07 ± 0.62	0.002
29	72	85.0	2.64	0.40 ± 0.02	0.50 ± 0.01	57.8	73.9	1.99 ± 0.41	<0.001
30	73	88.5	6.22	0.97 ± 0.03	0.86 ± 0.03	23.0	67.5	−2.23 ± 1.12	0.055
31	88	94.9	5.23	0.90 ± 0.02	0.99 ± 0.02	27.8	73.0	1.35 ± 0.53	0.012
32	88	93.9	5.50	0.92 ± 0.04	0.76 ± 0.03	58.2	48.7	−2.16 ± 0.91	0.029
33	67	94.4	2.21	0.47 ± 0.02	0.69 ± 0.02	68.1	48.7	2.11 ± 0.40	<0.001
34	70	75.3	6.85						
35	70	79.8	6.12	1.04 ± 0.05	0.77 ± 0.07	23.1	12.1	−7.26 ± 2.04	0.016

^1^Editing to insure linearity of growth during the test period resulted in some contemporary groups being reduced to fewer than four animals, in which case this analysis was not attempted.

It was therefore concluded that a 35-day test period with (daily) partial body weights was insufficient to characterize ADG during the postweaning period. Thus, a second test period of approximately 50 days was evaluated. As before, the 50-day tests were evaluated with and without the quality control restriction of linear ADG in partial body weight. When the ADG in partial body weight of each animal was not edited for linearity, the first 50 days of the test period explained across contemporary groups, on average, 82.7 ± 2.7% of the variation in ADG over the full test period. The latter portion of the test explained an average of 25.9 ± 3.3% providing additional (*P* < 0.05) information informing the full-test ADG in 22 of the 35 contemporary groups. With each animal in the data set having an *R*^2^ value greater than 0.9 for the linear regression of partial body weight on days on test resulted in coefficients of determination attributable to ADG from the initial and final portions of the test of 84.0 ± 3.3% and 27.4 ± 4.0%, respectively. The latter portion of the test provided significant (*P* < 0.05) information informing the full-test ADG in 13 of these 30 contemporary groups. Without and with having edited the data from each animal for linearity of growth during the entire test period, the corresponding average deviations of the predicted final partial body weight from its observed value averaged −1.83 ± 1.22 kg (*P* = 0.15) and −1.13 ± 0.94 kg (*P* = 0.24), respectively. Based on these results, it was concluded that the latter portion of the test period still contributed significant information to the prediction of full-test ADG. However, in an analysis that included all contemporary groups fitted as fixed effects, ADG in the first 50 days of the test period explained 80.0% of the within contemporary group variance with the latter portion of the test explaining only an additional 2.0%. In evaluating a 43 day test, ADG in the first part of the test period explained 68.2% of the within contemporary group variance, while ADG from the latter half of the test period explained an additional 8.5% of the within contemporary group variance. Thus, based on the pooled analysis, a test period of 50 days may be deemed adequate while a test period of 43 days would not.

The minimum length of the test period is not entirely a statistical consideration relative to the test itself. If the goal of the postweaning gain test is to obtain the most precise estimate of ADG possible, then a lengthy test period is preferable. Because the animals on test are likely to vary in age, a longer test would provide a better opportunity to evaluate efficiency at a constant age or degree of maturity through statistical adjustment to the appropriate endpoint. However, there are potential economic and experimental benefits from a shorter test period (Manafiazar et al., 2017). A shorter test period has the advantage of increasing the chance that growth is linear over the duration of the test. It is recommended that the prescribed length of test should depend on the anticipated use of the resulting data. In the context of genetic evaluations and many experiments, power of the test can be increased and additional accuracy may result from testing more animals, even if the individual measurements themselves are less precise (Lush et al., 1928; Manafiazar et al., 2017). Thus, a test of 50 days may be suitable for many purposes. However, as the postweaning gain test is shortened, the impact of any inaccuracy in the prediction of body weight affecting ADG may be magnified if the body weight records early and late in the test are not similarly affected.

## CONCLUSION

The work that has been reported herein is specific to one proprietary system and probably should not be extrapolated to competing technologies since differences in underlying algorithms used in the data capture and analysis are likely to exist. With any technology the individual researcher/bull test operator needs to be cognizant of potential for error in measures of body weight. Some users of the partial body weight technology are likely to lack the statistical expertise to determine the length of test that is desirable for their specific application. When the capacity of the facilities is the limiting constraint on the number of animals that can be tested, a 50-day test and testing more animals is likely result in greater power of the test for the hypothesis of interest. A shorter test period may also reduce the per animal cost of testing. However, if the number of animals that are available to test is the limiting factor, then a longer test is probably preferable. Predicting full body weight from partial body weight is likely to have acceptable accuracy in most applications, recognizing that there will be some degree of prediction error.
